# Performance enhancement at the cost of potential brain plasticity: neural ramifications of nootropic drugs in the healthy developing brain

**DOI:** 10.3389/fnsys.2014.00038

**Published:** 2014-05-13

**Authors:** Kimberly R. Urban, Wen-Jun Gao

**Affiliations:** ^1^Department of Psychology, University of DelawareNewark, DE, USA; ^2^Department of Neurobiology and Anatomy, Drexel University College of MedicinePhiladelphia, PA, USA

**Keywords:** methylphenidate, modafinil, ampakine, cognitive enhancement, synaptic plasticity, brain development

## Abstract

Cognitive enhancement is perhaps one of the most intriguing and controversial topics in neuroscience today. Currently, the main classes of drugs used as potential cognitive enhancers include psychostimulants (methylphenidate (MPH), amphetamine), but wakefulness-promoting agents (modafinil) and glutamate activators (ampakine) are also frequently used. Pharmacologically, substances that enhance the components of the memory/learning circuits—dopamine, glutamate (neuronal excitation), and/or norepinephrine—stand to improve brain function in healthy individuals beyond their baseline functioning. In particular, non-medical use of prescription stimulants such as MPH and illicit use of psychostimulants for cognitive enhancement have seen a recent rise among teens and young adults in schools and college campuses. However, this enhancement likely comes with a neuronal, as well as ethical, cost. Altering glutamate function via the use of psychostimulants may impair behavioral flexibility, leading to the development and/or potentiation of addictive behaviors. Furthermore, dopamine and norepinephrine do not display linear effects; instead, their modulation of cognitive and neuronal function maps on an inverted-U curve. Healthy individuals run the risk of pushing themselves beyond optimal levels into hyperdopaminergic and hypernoradrenergic states, thus vitiating the very behaviors they are striving to improve. Finally, recent studies have begun to highlight potential damaging effects of stimulant exposure in healthy juveniles. This review explains how the main classes of cognitive enhancing drugs affect the learning and memory circuits, and highlights the potential risks and concerns in healthy individuals, particularly juveniles and adolescents. We emphasize the performance enhancement at the potential cost of brain plasticity that is associated with the neural ramifications of nootropic drugs in the healthy developing brain.

## Introduction

Cognitive enhancement, and the ethical considerations that go along with it, is one of the hottest current topics in the neuroscience community. Humans have sought substances to improve our cognitive function for centuries, from ancient civilizations using hallucinogens in an attempt to raise their consciousness to commune with their gods, to the rise of coffee, to the more recent development of drugs such as stimulants and glutamate activators. Some might argue, therefore, that seeking to improve ourselves is a human trait, and therefore cognitive enhancement is nothing more than our application of new scientific approaches to meet our age-old desire for self-improvement and development. However, others argue that artificially enhancing one’s cognitive abilities is unfair and gives an unbeatable advantage to the richer populations who will have more ready access to the drugs (Butcher, 2003; Cakic, 2009). The issue of cognitive enhancement has even been likened to the steroid debate in sports (Cakic, 2009). There are many comprehensive reviews and articles published on the ethical concerns of cognitive enhancement; however, literature on the safety of consuming these drugs in youth is starkly lacking despite the significant increase in teen misuse and abuse of stimulants reported in a recent national study (Goldberg, 2013). Therefore, for the purpose of this review, we will concentrate on examining potential neurobiological ramifications of the popular cognitive enhancers, and highlight recent data on these drugs’ actions in developing brains. It is likely that a large proportion of the population is exposed to cognitive enhancing drugs and pressure to take them may be especially high among college and high school students; these individuals are facing more stringent college and graduate school acceptance criteria, limited job pools and an ever-increasing pressure to perform better and better if they hope to succeed (Goodman, 2010; Franke et al., 2011; Lynch et al., 2011). However, individuals in this population may be the ones most likely at risk for potential neurological consequences, due to their still-developing brains. We express regret that we are not able to cite many other good articles due to the topic specificity and sparsity of existing research; however, interesting information on cognitive enhancers that was outside the scope of this review can be found in these additional references (Dresler et al., 2013; Pang and Hannan, 2013; Ragan et al., 2013; Madan, 2014).

## Methylphenidate and the developing brain

**Figure 1:**
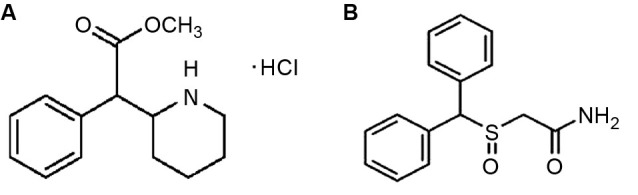
**(A)** Chemical structure of methylphenidate (Ritalin©). **(B)** Chemical structure of modafinil (Provigil©). The drug bears a striking resemblance to methylphenidate and other stimulants.

One of the most popular drugs under consideration for cognitive enhancement was originally developed to treat attention deficit-hyperactivity disorder (ADHD). Methylphenidate (Ritalin©; MPH; Figure 1A) is currently the most commonly prescribed medications for the treatment of ADHD (Challman and Lipsky, 2000; Spiller et al., 2013). MPH is a psychostimulant, related to amphetamine and cocaine and exerts its effects by blocking the transporters that reuptake dopamine and norepinephrine into the presynaptic neuron following their release; thus, it increases the levels or prolongs the availability of these neurotransmitters in the synapses to exert effects on postsynaptic neurons (Kuczenski and Segal, 2005).

However, a large proportion of literature on the safety and efficacy of MPH comes from studies performed on normal, healthy adult animals, as there is currently no sufficiently reliable animal model for ADHD. Several decades ago, studies began emerging that suggested that reduced hyperactivity and impulsivity in stimulant-treated ADHD patients were not “paradoxical” effects, but in fact also occurred in healthy individuals given the same doses (Rapoport et al., 1978, 1980). More recent MPH studies in both humans and rats have found that low doses of MPH that correspond to those given to ADHD patients in the clinic appear to enhance prefrontal-dependent functions and cognition in much the same way in healthy humans and rats as they do in ADHD patients and disease model rat strains (Mehta et al., 2001; Askenasy et al., 2007; Dow-Edwards et al., 2008; Agay et al., 2010; Linssen et al., 2012). These facts led to not only the acceptance of MPH study in normal subjects, but also the consideration of the medication as a cognitive enhancer.

The vast majority of studies on the cognitive enhancing effects of MPH and its effects on the normal brain have been performed in adult animals or humans. Higher doses (doses greater than those given to treat ADHD; 5–10 mg/kg intraperitoneal in rats) increase locomotor activity and impair attention and performance on prefrontal cortex-dependent cognitive tasks; however, lower doses (doses equivalent to the range given to ADHD patients; 0.5–2 mg/kg intraperitoneal in rats) improve cognitive performance and reduce locomotor activity in healthy individuals (Mehta et al., 2001). Likewise, lower doses of MPH (0.25–1 mg/kg, intraperitoneal, i.p.) in normal adult rats resulted in increased performance on attention tasks along with no effect on locomotor activity, while higher doses impaired performance and caused hyperactivity; doses beyond 10 mg/kg resulted in “stereotypes” (repetitive, fine motor movements similar to the tics seen in disorders like Tourette’s syndrome) (Mehta et al., 2001). The low doses of MPH result in slight increases in dopamine and norepinephrine selectively in the prefrontal cortex, while not affecting other brain regions (Berridge et al., 2006). This allows for improvements in executive control and working memory (WM) without inducing locomotor activity or stereotypes.

**Figure 2:**
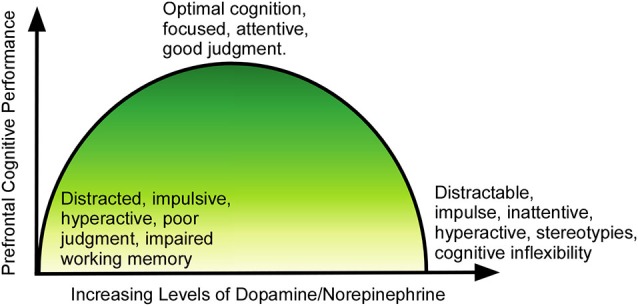
**Relationship of dopamine/norepinephrine to prefrontal function.** At lower than optimal levels, the PFC is underactive, and the individual suffers from symptoms of ADHD (impulsivity, poor judgment, inattentiveness, motor hyperactivity). As levels rise, the function improves, until cognition and executive function reaches peak performance at optimal levels of dopamine/norepinephrine. As levels of the neurotransmitters continue to rise past the optimal point, cognition again becomes impaired, with the individual showing distractability, impulsivity, stereotypical behaviors and cognitive inflexibility.

However, the dangers of cognitive enhancement with stimulants like MPH lie in their potential effects on the regulation of dopamine and norepinephrine (Figure 2). At optimal doses, dopamine binds to higher-affinity D1-like receptors, and norepinephrine binds to α2 receptors, leading to an increase in prefrontal cortical signal-to-noise ratio and enhancing the flow of information and strengthening neuronal communication (Arnsten and Li, 2005). When the levels of dopamine and norepinephrine rise beyond the optimal levels, they begin to activate dopamine D2-class receptors and noradrenergic α1 and β receptors, which leads to weakening of the signal-to-noise ratio via activation of neurons that may not be involved in the current task (Arnsten and Li, 2005; Arnsten, 2009b). This nonspecific activation impairs attentional selectivity and results in a manifestation of locomotor hyperactivity, distractability and poor impulse control.

Levels of dopamine and norepinephrine in a normal, healthy brain are not universal and they may vary slightly over time within the same individual based on season, time of day, or activity (Otter and Nurmand, 1980; Petrović et al., 1980). Currently, there is no reliable method for determining optimal levels of these neurotransmitters in living human brains; thus, predicting how a certain dose of MPH will affect a particular person is largely an educated approximation. It is also possible that, although many studies found no overt cognitive differences between the effects of low-dose MPH on normal individuals and ADHD patients, molecular or cellular differences may exist that await detection by the development of more advanced technology. Thus, one must examine the research on MPH as a cognitive enhancer and studies using normal individuals with caution.

MPH is currently most often abused and sold on the black market among adolescents, particularly in high schools and on college campuses (Goodman, 2010; Franke et al., 2011). Students look for the medication when they have exams, or need to stay awake for long hours, in order to boost their energy and memory. This rather commonplace usage among adolescents is particularly frightening in light of the developmental timeline of the prefrontal cortex. This brain region, the center of control of judgment, behavioral inhibition and emotion, WM, logical thinking and decision making, does not finish developing until young adulthood; in humans this falls around the end of the second decade or the beginning of the third decade of life (Casey et al., 2008). During adolescent development, the levels of norepinephrine and dopamine surge and wane to allow for maturation of the executive control and reward pathways (Kanitz et al., 2011). Introducing a substance that alters dopamine and norepinephrine levels, such as MPH, might disrupt the maturation of the prefrontal cortex and have lasting behavioral consequences.

Indeed, research has recently begun to shift toward understanding MPH’s actions in a juvenile brain. These pioneering studies have yielded striking results, indicating that early life treatment with MPH may alter circadian rhythms, induce anxiety that persists into adulthood, and even impair object-recognition memory (Lee et al., 2009; Algahim et al., 2010). However, many of the studies have not been particularly stringent in their dosing regimens, and the reader must examine the amount of drug used in each study very carefully. In adult rats, a therapeutic, clinically-relevant dose of MPH is one that produces blood plasma levels of 8–40 ng/dL; this appears to be in the range of 0.25–1 mg/kg given in an intraperitoneal injection (i.p.) (Berridge et al., 2006). We have recently completed several studies examining the effects of a low therapeutic dose (1 mg/kg, i.p.) on juvenile rats. We reported that a single dose of MPH resulted in significant depression of neuronal excitability and synaptic transmission in the prefrontal cortex; treatment with a chronic regimen of 3 weeks resulted in even further depression (Urban et al., 2012). In adult rats, however, the same low dosage increased neuronal activity (Urban et al., 2012). These results suggest that there is an age-dependent difference in MPH’s actions, and that in healthy juveniles and adolescents, the doses previously thought to be therapeutic and cognitively enhancing may in fact be inducing excessive levels of dopamine and norepinephrine and in fact impairing certain aspects of cognition. Further supporting this theory, we discovered that the depression of neuronal activity was due, at least in part, to activation of a channel known as the hyperpolarization-activated non-specific cation channel (HCN; Urban et al., 2012). The HCN channel allows for flow of positively-charged ions, particularly potassium, out of the neuron, lowering its voltage potential and making it harder for the neuron to fire action potentials. The HCN channel is also known to be activated by a hyperdopaminergic state; thus, its role in juvenile treatment with MPH suggests that the dosage is inducing excessive dopamine, and possibly norepinephrine as well (Arnsten, 2009a).

One important unique property of the prefrontal cortex is its high level of plasticity, allowing for executive functions like WM and active decision-making; this plasticity may be a product of the slow maturation of this region (Jernigan et al., 1991; Kuboshima-Amemori and Sawaguchi, 2007; Spencer-Smith and Anderson, 2009; Newman and McGaughy, 2011; Teffer and Semendeferi, 2012; Selemon, 2013). Plasticity is controlled by levels of α-amino-3-hydroxy-5-methyl-4-isoxazolepropionic acid (AMPA) receptors and n-methyl-d-aspartate (NMDA) receptors. NMDA receptors contain two NR1 subunits with a combination of either NR2A or NR2B; NR2B conveys slower kinetics to the channel, allowing it to remain partially open during multiple stimulations (Cull-Candy et al., 2001). This property results in summation of responses and the continuation of the neural activity briefly after input has stopped, which is thought to be the neuronal correlate of WM (Wang et al., 2008, 2013). In most cortical brain regions, the ratio of NR2B/NR2A is high at birth, and declines over development; however, in prefrontal cortex it remains high (Wang et al., 2008). This allows for retention of plasticity throughout life, allowing the prefrontal cortex to continually adapt to incoming information and adjust behavioral output. We recently discovered that low dose (1 mg/kg, i.p.) treatment of juvenile rats with MPH induced a selective decrease in the levels of NR2B without affecting NR2A subunits (Urban et al., 2013). This finding supports our theory that the juvenile brain may be hypersensitive to dopamine levels; excessive levels of dopamine induce internalization of NR2B receptors via activation of glycogen synthase kinase (GSK)-3β, which causes phosphorylation of β-catenin, disrupting the β-catenin-NR2B interaction that stabilizes the NR2B subunit (Li et al., 2009). With β-catenin unbound, the NR2B subunits become targeted for internalization.

What do our findings mean for the healthy adolescent taking MPH? The prefrontal cortex’s uniquely high levels of NR2B subunits throughout life impart the ability of the neurons to summate responses to incoming stimuli, resulting in the short-term potentiation of neural activity necessary for WM; thus, decreasing the levels of NR2B in prefrontal cortex leads to a reduction in the summation, which should impair WM (Wang et al., 2008, 2013; Urban et al., 2013). However, long-term potentiation (LTP) was found to be enhanced following juvenile treatment with MPH (Urban et al., 2013). The exact roles of NR2A versus NR2B receptor subunits in LTP regulation in the prefrontal cortex are not well understood, but it is currently believed that the direction of plasticity in prefrontal cortex (potentiation or depression) is dependent on the ratio of NR2A/NR2B, rather than exact levels of each subunit (Massey et al., 2004; Xu et al., 2009; Foster et al., 2010). Thus, reducing NR2B levels without altering NR2A levels, as was seen following juvenile MPH treatment, was enough to alter the direction of PFC long-term plasticity (Urban et al., 2013). The behavioral ramifications of altering LTP and long-term depression (LTD) in the prefrontal cortex are unclear, as it is not known exactly what LTP is representing in this region. However, it has been hypothesized that, if short-term potentiation is a cellular constituent of WM, then LTP might be a marker of sustained attention and long-term memory consolidation. Thus, perhaps treatment of the healthy juvenile brain with these low doses of MPH results in impaired WM and behavioral flexibility, but enhanced sustained attention and long-term memory? If this is the case, it could indicate that MPH-treated children who do not in fact have ADHD would appear successfully treated in a classroom setting—these children would be paying attention to the teacher, less hyperactive and learning might improve. However, stringent testing of their behavioral flexibility and WM might reveal subtle deficits that may affect their lives. For example, behavioral flexibility is needed for driving an automobile—the driver must be able to quickly shift attention from the road, to road signs, other approaching vehicles, back to the road and so on. Rigid attention and lack of flexibility could potentially result in inattentive or distracted driving. Behavioral flexibility is also a critical component of interpersonal skills; one must be able to adapt to different individuals and, in a work setting, shift plans and roles within the group in order to achieve the goal. Again, behavioral and cognitive inflexibility could impair the individual’s function at their job and lead to reduced pay, unemployment or disciplinary action. Finally, behavioral flexibility is a critical component of resisting and recovering from drug abuse. Kalivas and Volkow identified alterations in glutamatergic signaling that result in an inability to alter one’s behavior in cocaine addicts (Kalivas and Volkow, 2005, 2011; Kalivas et al., 2005). MPH has been shown to reduce the likelihood of later drug abuse in individuals diagnosed with ADHD, but, as the drug appears to affect glutamatergic signaling, it could potentially result in similar behavioral rigidity and lead to an increased likelihood for obsessive-compulsive or addictive behaviors (Newman and McGaughy, 2011).

## Modafinil—potential for stimulant-like effects?

MPH’s effects on brain development are surely interesting and potentially frightening; however, it is not the only cognitive enhancing medication that alters dopamine and glutamate transmission. Another cognitive enhancer that has begun to receive attention in the scientific community is modafinil (Provigil©), which bears a striking structural resemblance to methylphenidate (MPH) and other stimulants (Figure 1B). Originally developed in France in the 1970s, modafinil elevates hypothalamic histamine levels, but also appears to have a striking affinity for cell surface dopamine transporters (Engber et al., 1998; Ishizuka et al., 2008; Zolkowska et al., 2009). Its exact mechanism of action remains under debate, although arguments have been made both for its performing more as a wakefulness-promoting reagent via the hypocretin/orexin system of the hypothalamus, and as a classical psychostimulant via its blockade of the dopamine reuptake inhibitor (Ishizuka et al., 2003; Zolkowska et al., 2009). However, modafinil still promotes wakefulness in orexin knockout mice, so it appears that the orexin system is not required for therapeutic benefits (Willie et al., 2005). Whatever the mechanism, or mechanisms, of action may turn out to be, modafinil is currently a heavily studied drug with multiple uses. It is currently approved by the US Food and Drug Administration (FDA) for the treatment of narcolepsy, shift-work disorder and obstructive sleep apnea (Erman and Rosenberg, 2007; Cephalon, 2013). It has been shown to reduce jet lag and improve mood among shift workers, who often struggle with depression and chronic fatigue, forgetfulness and general cognitive impairments brought on by their work hours not allowing for a steady sleep-wake cycle (O’Connor, 2004; Hart et al., 2006). Modafinil has also been studied as an alternative to amphetamines for military usage—the military provides stimulants to soldiers in sleep-deprivation or high stress situations that require extreme alertness for long stretches of time. It is currently approved for Air Force missions in the US, and is also used in the UK and India (Taylor and Keys, 2003; Wheeler, 2006; Sharma, 2011).

Although modafinil is considered a first-line therapy for excessive daytime sleepiness (EDS) associated with narcolepsy in adults; it is also widely used in the treatment of EDS in children (Ivanenko et al., 2003; Sullivan, 2012). Caution is again the rule, especially at younger ages, due to reports of serious adverse events (such as tachycardia, insomnia, agitation, dizziness and anxiety) in elevated modafinil doses (Spiller et al., 2009), and in fact, the manufacturer recommends against use of modafinil in younger children. Despite these reports, modafinil is FDA-approved for use in children over age 16 years (Sullivan, 2012).

The usefulness of modafinil in improving alertness and wakefulness in non-sleep-deprived, healthy individuals, and its military involvement, has led to the consideration of the drug as a cognitive enhancer (Turner et al., 2003; Baranski et al., 2004; Randall et al., 2005b). Most studies agree that modafinil induces improvements in pattern recognition memory, digit span recall and mental digit manipulation (performing addition/subtraction/multiplication in one’s mind), but the effects on spatial memory, attention and other aspects of executive function are more ambiguous, and appear to depend on the baseline performance of the individual in question (Turner et al., 2003; Baranski et al., 2004; Müller et al., 2004; Randall et al., 2005b). In a study of healthy student volunteers, modafinil improved target sensitivity in a rapid visual information processing (RVIP) task, and speed of color naming and drawing, but only in individuals with a “low” (mean 106 + 6) IQ; it had no significant effect on individuals with “higher” (mean 115 + 5) IQs (Randall et al., 2005a). In rats, these results are replicated, with low responding rats showing improvement on stop-signal reaction time tests after modafinil; higher performing rats showed no improvement (Eagle et al., 2007). Interestingly, MPH also shows sensitivity to baseline performance; many studies have indicated that MPH induces greater improvement in low-performing individuals than in higher performing individuals, and in some cases may actually cause deficits in higher performers (Eagle et al., 2007; Finke et al., 2010).

A recent study conducted in healthy human subjects reported that modafinil differs from other arousal-enhancing agents in chemical structure, neurochemical profile, and behavioral effects (Rasetti et al., 2010). Unlike most functional neuroimaging studies that focused on the effect of modafinil only on information processing underlying executive cognition, this study examined the effect of modafinil on neural circuits underlying affective processing and cognitive functions. They underwent blood-oxygen-level dependent (BOLD) functional magnetic resonance imaging (MRI, or functional MRI, fMRI) while performing an emotion information-processing task that activates the amygdala and two prefrontally dependent cognitive tasks—a WM task and a variable attentional control (VAC) task. BOLD fMRI revealed significantly decreased amygdala reactivity to fearful stimuli on modafinil compared with the placebo condition. During executive cognition tasks, a WM task and a VAC task, modafinil reduced BOLD signal in the prefrontal cortex and anterior cingulate. This study suggested that modafinil in low doses has a unique physiological profile compared with stimulant drugs: it enhances the efficiency of prefrontal cortical cognitive information processing, while dampening reactivity to threatening stimuli in the amygdala, a brain region implicated in anxiety (Rasetti et al., 2010).

The baseline performance sensitivity, and dopamine reuptake transporter affinity, indicates that modafinil could induce similar effects on the brain as psychostimulants like MPH. If this is the case, cause for concern arises when modafinil is considered as a cognitive enhancer in adolescents and young adults. To enlist in the Air Force, where modafinil is currently in use for pilots, one must be between 17–27 years of age (U. S. Air Force, 2013). The prefrontal cortex, under tight regulation by levels of dopamine and norepinephrine, and the brain’s main center of attention and executive processing, does not finish development until the late 20’s to early 30’s for humans; thus, young pilots may be at risk for modafinil inducing excessive levels of dopamine in this brain region (Casey et al., 2008). One can expect that the potential ramifications of modafinil use in healthy young adults and teenagers would be similar to those seen in juvenile/adolescent use of MPH (Urban et al., 2012, 2013). Thus, modafinil could induce changes in plasticity or behavioral rigidity, and potentially damage WM, logical thinking and decision making. It has been reported that prolonged wakefulness induces experience-dependent synaptic plasticity in mouse hypocretin/orexin neurons (Rao et al., 2007). Specifically, acute and chronic prolonged wakefulness in mice induced by modafinil treatment produced LTP of glutamatergic synapses on hypocretin/orexin neurons in the lateral hypothalamus, a well-established arousal/wake-promoting center. A similar potentiation of synaptic strength at glutamatergic synapses on hypocretin/orexin neurons was also seen when mice were sleep deprived for 4 h. These results indicate that synaptic plasticity due to prolonged wakefulness occurs in circuits responsible for arousal and may contribute to changes in the brain of animals experiencing sleep loss. It is therefore likely that misuse and abuse of modafinil in the teens will eventually result in brain plasticity, especially brain regions related to sleep and motivation such as hypothalamus and dopamine-rich prefrontal cortex, hippocampus and nucleus accumbens. Future studies will need to address these shortcomings in order to determine the safety and efficacy of modafinil as a true cognitive enhancer. Recent reviews proposed some interesting mechanisms that may explain the likelihood of cognitive enhancement (Lynch et al., 2011; Roesler and Schröder, 2011; Lynch and Gall, 2013) but experiments are warranted for further exploration. The current research is contradictory in that some studies have noted clear improvements in sustained attention in humans, while others have failed to find any effect of the drug (Turner et al., 2003; Randall et al., 2005b ). Similar discrepancies can be found in rodent studies; however, more recent studies are pointing to the possibility that modafinil selectively enhances WM without affecting consolidation of memories into long-term storage (Béracochéa et al., 2002; Turner et al., 2003; Müller et al., 2004; Randall et al., 2005b; Minzenberg and Carter, 2008). These studies are interesting, and suggest striking utility of modafinil as a cognitive enhancer; however, they have been performed on adult humans and rodents. MPH has also been shown in studies of healthy adults and children with ADHD to apparently enhance WM (Mehta et al., 2004; Pietrzak et al., 2006; Kobel et al., 2009; Marquand et al., 2011), yet recent juvenile rat studies suggest that in a healthy, developing brain, the drug might actually impair WM at low doses thought to be clinically relevant, i.e., doses that produce blood plasma levels of 8–40 ng/dL (Urban et al., 2013). Modafinil’s profile by showing improvements in WM in healthy adults and sleep-deprived individuals (the population the drug was originally developed for) is analogous to MPH promoting improvements for healthy adults and children with ADHD; since both drugs appear to affect dopamine levels through blockade of the reuptake transporters, and alter glutamate signaling, it stands to reason that they could result in similar effects on WM in healthy, juvenile brains. Thus, modafinil at certain doses might cause a reduction in NMDA receptor levels, impairments in short-term plasticity and alterations in long-term plasticity much as MPH does (Urban et al., 2013). Future studies of modafinil as a cognitive enhancer should examine this possibility, and establish whether the drug shows an age- and dose-dependent profile of effects like the classic psychostimulants.

## Ampakines—drugs for treatment of Alzheimer’s disease—turned cognitive enhancers

The final classes of medications we will discuss in this review are the ampakines, which also have potential for significant effects on the developing glutamatergic system. Ampakines are a class of drugs that bind to the glutamatergic AMPA receptor, enhancing its activity by slowing deactivation and attenuating desensitization of AMPA receptor currents, increasing synaptic responses and enhancing LTP (Arai and Kessler, 2007). AMPA receptors are critically involved in regulating cortical plasticity; trafficking of AMPA receptors to the synapse is crucial for maintenance of excitability that leads to LTP (Malinow and Malenka, 2002; Huganir and Nicoll, 2013). However, there is more to the story of how AMPA regulates excitability; it does not function alone in the process. A second class of ionotropic glutamate receptors, NMDA receptors, actually trigger the induction of LTP; however, these receptors are normally blocked by magnesium at resting membrane potentials (Dingledine et al., 1999; Cull-Candy et al., 2001; Paoletti et al., 2013). Activation of AMPA receptors induces EPSCs, which depolarize the neuron and remove the magnesium block of NMDA, allowing for the induction of LTP. Then, NMDA receptors increase trafficking of more AMPA receptors to the synapse, maintaining the LTP (Lu et al., 2001; Paoletti et al., 2013). No ampakines are currently FDA approved, but they are being investigated as treatments for Alzheimer’s senility, Parkinson’s disease, ADHD, Rhett syndrome, schizophrenia, depression, autism, and Angelman syndrome (AS; Goff et al., 2001; Arai and Kessler, 2007; Ogier et al., 2007; Wezenberg et al., 2007; Simmons et al., 2009; Baudry et al., 2012; Silverman et al., 2013). However, they’ve also shown effectiveness at improving memory and cognition in healthy adult volunteers and rats (Ingvar et al., 1997; Hampson et al., 1998; Lynch and Gall, 2006; Wezenberg et al., 2007). Ampakines are also being studied by the US military for use as cognitive enhancers and alertness promoters for soldiers in high-stress extended combat situations; the lack of central nervous stimulation (such as would occur with modafinil, amphetamines or MPH make the ampakines very attractive (Saletan, 2008). Although ampakines have few adverse effects at therapeutically relevant concentrations and protect neurons against neurotoxic insults in adults (Arai and Kessler, 2007), the ampakine faramptor can cause headache, somnolence and nausea (Wezenberg et al., 2007).

While the ampakines represent perhaps the most promising group of pharmaceuticals for low-risk cognitive enhancement, as well as a potential relief for sufferers of psychiatric illnesses, they are likely not without danger to teens, adolescents, and young adults. First, very little is known about these drugs; the only example to reach human clinical trials is Cortex Pharmaceuticals’ CX-717, which was evaluated in Phase I for the treatment of Alzheimer’s disease; histological damage was seen in animal studies but Cortex claimed this was an artifact of tissue fixation (Stoll and Griesel, 2007). The FDA denied the application, and CX-717 approval halted. None of the other ampakines is known to currently be in human trials, so little can be proven about their efficacy or safety in healthy individuals. However, we can speculate based on knowledge of plasticity and the glutamate system.

The first concern when stimulating glutamate transmission in the brain is the potential for excitotoxicity. Glutamate toxicity generally occurs when excess glutamate storms the AMPA and NMDA receptors, causing a mass influx of calcium. This excess calcium in the cells activates a number of enzymes like proteases and phospholipases, which induce damage to organelles, the cell membrane, and DNA (Manev et al., 1989; Ankarcrona et al., 1995). However, activating AMPA receptors directly would cause a similar mass influx of cations and could also induce excitotoxicity. A recent study reported that ampakines promote spine actin polymerization, LTP, and learning in a mouse model of AS (Baudry et al., 2012). AS is a neurodevelopmental disorder largely due to abnormal maternal expression of the UBE3A gene leading to the deletion of E6-associated protein. AS subjects have severe cognitive impairments for which there are no therapeutic interventions. Mouse models (knockouts of the maternal UBE3A gene: “AS mice”) of the disorder have substantial deficits in LTP and learning. Baudry et al reported that ampakine CX929 significantly enhanced LTP and notably, reduced dendritic spine abnormality and learning impairments (Baudry et al., 2012). This minimally invasive drug treatment is certainly promising for AS, and probably other neurodevelopmental disorders such as fragile X syndrome and autism (Rueda et al., 2009; Silverman et al., 2013) as well. However, such a magnitude of effects on synaptic plasticity and dendritic spine integrity also raises serious concern for immature brains of young children using ampakines as cognitive enhancers. It is not difficult to imagine that ampakines would have similar effects on the synaptic transmission and neuronal communication in the normal brain, eventually eliciting brain plasticity in the regions that are associated with emotional and affective functions. This could potentially lead to poor emotional regulation and impaired behavioral inhibition if plasticity is excessive and unregulated. Indeed, one of the important mechanisms by which the brain connections are maintained and tuned is through synaptic pruning, whereby highly active synapses are strengthened and less active synapses are removed through axon retraction (Luo and O’Leary, 2005; Gazzaniga and Mangum, 2009; Kolb et al., 2012). At first thought, heightened plasticity might seem to be a benefit—translating to faster learning and improved cognitive function; however, the excessive plasticity could also lead to high activity in all synapses and therefore reduce synaptic pruning. Impairments in synaptic pruning have in fact been associated with autistic spectrum disorders (Belmonte et al., 2004). The excessive connectivity leads to a heightened overall brain activation but the reduction in selectivity of activation is such that the signal-to-noise ratio is greatly lowered (Belmonte, 2000; Belmonte and Yurgelun-Todd, 2003). Thus, one can clearly see the potential dangers associated with unregulated plasticity, and how ampakines (which strengthen synapses and heighten plasticity by promoting dendritic spine growth) might lead to autism-like syndromes.

Although no studies have yet noted this in humans, doses of ampakines given to humans thus far have been tightly controlled. If the drug became available as a cognitive enhancer, or reached the black market, individuals could easily exceed safe doses and suffer neuronal damage from glutamate toxicity. Furthermore, the main purported therapeutic action of the ampakines is an alteration of plasticity; they are known to lower the threshold for induction of LTP and also increase the magnitude of LTP achieved (Lynch and Gall, 2006). While this alteration of plasticity may improve many aspects of learning and cognition, such as alertness, enhancement of LTP will likely come with a concomitant decrease in the opposite direction of plasticity, i.e., LTD. LTD is crucial for formation of spatial maps, and might play a role in cerebellar motor learning as well (although studies of motor performance after LTD impairment have been somewhat contradictory) (Aiba et al., 1994; Manahan-Vaughan, 2005; Kemp and Manahan-Vaughan, 2007). Thus, shifting plasticity in favor of LTP could lead to impairments in spatial memory and perhaps motor function. Careful determination of a dose-response curve, excitotoxic effects and species differences in metabolism/reaction to ampakines will need to be completed in the future in order to determine their true utility as cognitive enhancers.

## Conclusion and future perspective

In this review, we have examined three major pharmaceuticals under consideration as cognitive enhancers—MPH, modafinil and the ampakines. We have reported striking and deeply concerning effects of clinically relevant doses of MPH on the juvenile prefrontal cortex function and plasticity, compared them to the potential ramifications of modafinil treatment, and suggested several potential risks of ampakine exposure in healthy individuals. It is clear from the current lack of research in the field that much work needs to be done in order to determine the safety of cognitive enhancers, particularly among adolescents, the population most likely to take advantage of these drugs should they become available. There is already a high demand on college campuses and in high schools for MPH; thus, many healthy adolescents and young adults are already being exposed to unregulated doses of this substance. Understanding the behavioral and functional ramifications in cellular and molecular changes in the yet immature brains is paramount to mitigating risks for potential brain plasticity and consequent emotional and behavioral changes (Urban and Gao, 2012, 2013).

It is currently unclear if the dose range of stimulants that translates to effective ADHD symptom alleviation and cognitive enhancement in the healthy adult will translate to the same behavioral effects in juveniles; however, our recent studies suggest that the juvenile brain is hypersensitive to the effects of MPH (Urban et al., 2012). Thus, even a low, purportedly clinically relevant dose is likely to cause excessive levels of dopamine and norepinephrine, and impair executive functions and WM. This excessive dopamine/norepinephrine is likely also a potential risk of juvenile treatment with modafinil. It is far less clear how the ampakines might affect juvenile brain function, but their effects on plasticity through the glutamatergic system warrants further exploration. The desire for development of cognitive enhancing substances is unlikely to diminish with time; it may represent the next stage in evolution—man’s desire for self-improvement driving artificial enhancement of innate abilities. It is therefore the responsibility of scientists and the medical community to stringently evaluate and research each new candidate substance, furthering our understanding of the brain in the process. Perhaps most importantly, the role of age and developmental stage in individual responses to cognitive enhancing substances needs to be thoroughly examined. Juvenile metabolic rates compared to adult are not clear in humans or rodent models; the dose-response curve for juveniles compared to adults for MPH, modafinil and the ampakines, as well as many other psychoactive medications, has not been examined. Finally, a potential long-term ramification of early life exposure of the healthy juvenile brain to these substances is only a very recent emerging topic of research, and much care needs to be taken to answer the questions expediently. Cognitive enhancement is no longer a scientific fiction; we must consider the unique dynamics of the developing brain and proceed cautiously until thorough safety and efficacy parameters have been established.

## Conflict of interest statement

The authors declare that the research was conducted in the absence of any commercial or financial relationships that could be construed as a potential conflict of interest.
